# Rapid Crown Root Development Confers Tolerance to Zinc Deficiency in Rice

**DOI:** 10.3389/fpls.2016.00428

**Published:** 2016-03-31

**Authors:** Amrit K. Nanda, Matthias Wissuwa

**Affiliations:** Crop, Livestock and Environment Division, Japan International Research Center for Agricultural SciencesTsukuba, Japan

**Keywords:** zinc, rice, *Oryza sativa*, crown root, development, translocation, efficiency, tolerance

## Abstract

Zinc (Zn) deficiency is one of the leading nutrient disorders in rice (*Oryza sativa*). Many studies have identified Zn-efficient rice genotypes, but causal mechanisms for Zn deficiency tolerance remain poorly understood. Here, we report a detailed study of the impact of Zn deficiency on crown root development of rice genotypes, differing in their tolerance to this stress. Zn deficiency delayed crown root development and plant biomass accumulation in both Zn-efficient and inefficient genotypes, with the effects being much stronger in the latter. Zn-efficient genotypes had developed new crown roots as early as 3 days after transplanting (DAT) to a Zn deficient field and that was followed by a significant increase in total biomass by 7 DAT. Zn-inefficient genotypes developed few new crown roots and did not increase biomass during the first 7 days following transplanting. This correlated with Zn-efficient genotypes retranslocating a higher proportion of shoot-Zn to their roots, compared to Zn-inefficient genotypes. These latter genotypes were furthermore not efficient in utilizing the limited Zn for root development. Histological analyses indicated no anomalies in crown tissue of Zn-efficient or inefficient genotypes that would have suggested crown root emergence was impeded. We therefore conclude that the rate of crown root initiation was differentially affected by Zn deficiency between genotypes. Rapid crown root development, following transplanting, was identified as a main causative trait for tolerance to Zn deficiency and better Zn retranslocation from shoot to root was a key attribute of Zn-efficient genotypes.

## Introduction

Food security is defined as the access to sufficient, safe and nutritious food. To this date, it is estimated that approximately 795 million people in the world suffer from food insecurity, mostly in developing countries (Food and Agriculture Organization, 2015). As the world population is expected to reach 9 billion by 2050, the need to increase food production, in order to achieve food security, becomes more and more urgent. However, many external factors such as drought, flooding, salinity, and low soil fertility often prevent crops from reaching their true yield potential, creating yield gaps.

Rice (*Oryza sativa*) is one of the most important crop species and a major food staple in many developing countries, mainly in Asia, but increasingly so in Africa. At the same time, zinc (Zn) deficiency is the most widespread micronutrient disorder in rice (Nene, 1966; Yoshida and Tanaka, 1969; Yoshida et al., 1973). Zn is essential for plant growth and development, as it is involved in many physiological processes, including enzyme activation and protein synthesis (Marschner, 1995). Zn deficiency affects rice most severely during the seedling stage, following transplanting, when plant mortality may occur. Less severe symptoms include brown spots and discoloration of the leaves and reduced plant growth. Surviving plants typically recover partially around 5 weeks after transplanting, but delayed maturation and reduced yield are typical (Yoshida and Tanaka, 1969; Breemen and Castro, 1980). Although an adequate amount of Zn could be present in the soil, its availability to plants is dependent on soil conditions. Many factors, including high soil pH (>7) and high bicarbonate, as well as phosphate and organic matter content, contribute to sequestering Zn in the soil, resulting in rice Zn deficiency (Dobermann and Fairhurst, 2000). These conditions are most common in submerged soils, where rice is typically grown.

The effects of Zn deficiency in rice were first discovered in the 1960s (Nene, 1966; Yoshida and Tanaka, 1969) and an extensive study examining soil samples from 15 countries revealed that half of the farmed soils were low in Zn, with a quarter of them being severely Zn deficient (Sillanpää, 1990). The high frequency of Zn deficient soils highlights the importance of finding a solution to overcome this stress. One way to alleviate Zn deficiency is the addition of Zn fertilizers. However, this is not only costly, putting extra strain on poor farmers, but it is often inefficient, as the Zn will quickly form insoluble complexes in the soil following flooding (Johnson-Beebout et al., 2009). Another way to increase yield under Zn deficiency is to grow rice varieties that are more tolerant to this stress, also known as Zn-efficient varieties. Many efforts have been made in the last decades by the International Rice Research Institute (IRRI) and collaborators to explore rice germplasm collections, in order to identify and investigate plants with enhanced tolerance to Zn deficiency. An overview of the past screening trials in Zn deficiency highlights a wide variation of tolerance to Zn deficiency in the rice germplasm (Quijano-Guerta et al., 2002; Ismail et al., 2007).

Generally, tolerance to Zn deficiency can be attained through two mechanisms: efficient utilization and translocation of Zn within the plant, also termed Zn use efficiency (ZUE), and higher uptake of Zn by roots. The latter could further be subdivided into root size and root efficiency effects. Interestingly, a previous report found that Zn deficiency in rice associates more strongly with maintaining high Zn uptake rates than with ZUE (Wissuwa et al., 2006). More recent studies suggest that improved Zn uptake from Zn deficient soils is due to root exudates, such as siderophores and low molecular weight organic acids, capable of enhancing Zn solubility in soil (Zhang et al., 1991; Suzuki et al., 2008; Ptashnyk et al., 2011). It has also been demonstrated that Zn-efficient rice varieties are capable of maintaining crown root growth to a higher extent than Zn-inefficient varieties under Zn deficiency (Widodo et al., 2010; Rose and Wissuwa, 2012; Rose et al., 2013; Mori et al., 2016). However, whether that is a cause or a consequence of Zn-efficiency remains unclear.

Roots are an essential part of the plant as they take up water and nutrients from the soil, as well as keep the plant anchored. The rice root system is made up of an embryonic seminal root and post-embryonic crown roots, with lateral roots branching off from both of these (Hoshikawa, 1989). The examination of several rice mutants affected in crown root development has helped uncover some of the mechanisms regulating crown root development (reviewed in Itoh et al., 2005; Rebouillat et al., 2009; Coudert et al., 2010; Mai et al., 2014). For example, the rice *crown rootless* (*crl*) mutants develop no, or few, crown roots, with each *CRL* gene involved in different aspects of crown root development (Inukai et al., 2001b, 2005; Liu et al., 2005; Kitomi et al., 2008a,b, 2011). In the last decade, the impact of root size and architecture in determining tolerance to abiotic stresses, such as drought and nutrient deficiency, has been repeatedly highlighted (Lynch, 2007; Rebouillat et al., 2009; Rose et al., 2013; Wu and Cheng, 2014). For instance, a study identifying a key gene controlling rice root architecture, *DEEPER ROOTING 1* (*DRO1*), demonstrated that altering root growth angle, as to increase deep-rooting, was successful in conferring tolerance to drought (Uga et al., 2013). In the same way, *PHOSPHORUS STARVATION TOLERANCE 1* (*PSTOL1*), a receptor-like kinase, was found to enhance phosphorus uptake in rice plants grown on low phosphorus-containing soils, by increasing root growth (Gamuyao et al., 2012).

In this study, we investigated the effects of Zn deficiency on seedling crown root development in rice genotypes, previously reported to differ in crown root number and root size under Zn deficient field conditions (Widodo et al., 2010; Rose and Wissuwa, 2012; Rose et al., 2013; Mori et al., 2016). Specifically, we tested (i) whether Zn deficiency affects crown root initiation or emergence; (ii) whether crown root number differs constitutively between genotypes, or only in response to Zn deficiency; (iii) to what extent differences in internal ZUE or in Zn translocation are associated with genotypic differences in crown root development; and (iv) if the maintenance of crown root development observed for Zn-efficient genotypes is a cause or a consequence of Zn efficiency. For this purpose, seedlings of several Zn-efficient and inefficient genotypes were grown under different Zn supply, in field and hydroponic experiments. The use of seedlings rather than more mature plants was considered most relevant, since the ability of a newly transplanted seedling to outgrow Zn deficiency, will determine whether it will survive and the length of the developmental delay it will undergo, if any.

## Materials and Methods

### Plant Materials

Previously classified Zn-efficient and inefficient rice genotypes were selected for these experiments based on Zn efficiency experiments at IRRI or previously reported data. Zn-efficient IR55179-3B-11-3 (IR55179), RIL46 and Zn-inefficient IR26, IR64, IR74 were used (Bowen, 1986; Wissuwa et al., 2006; Wu et al., 2010). The reference variety Nipponbare was also included in this study.

### Field Study: Experiment 1

A field experiment was conducted at IRRI, Los Baños, Philippines, during the dry season (January–February) of 2015. Zn-efficient IR55179 and Zn-inefficient IR26 and IR64 genotypes were raised in a nursery bed for 3 weeks, before being uprooted and transplanted to a Zn-deficient paddy field (Wissuwa et al., 2007; Mori et al., 2016). The number of roots per seedling was counted just before transplanting and again at 3 and 7 days after transplanting (DAT). Whole plant dry weight was determined after drying at 70°C in an oven for several days.

### Hydroponics Studies: Experiments 2 and 3

Seeds from the different rice genotypes were surface sterilized and directly sown in seedling trays floating in 12 μM FeEDTA and 0.1 mM CaCl_2_ solution in 22 L boxes. Seeds were incubated in the dark for 3 days before exposing to 12/12 h light/dark cycles at 30/25°C, with approximately 50% relative humidity and 280 μmol m^-2^ s^-1^ light. After 1 week, the solution was changed to half-strength modified Yoshida solution without Zn (Yoshida et al., 1972), with the composition of full strength Yoshida being: 1.42 mM NH_4_NO_3_, 0.05 mM KH_2_PO_4_ × 2H_2_O, 0.5 mM K_2_SO_4_, 1 mM CaCl_2_ × 2H_2_O, 1 mM MgSO_4_ × 7H_2_O, 9 μM MnCl_2_ × 4H_2_O, 0.07 μM (NH_4_)_6_Mo_7_O_24_ × 4H_2_O, 18.5 μM H_3_BO_3_, 0.16 μM CuSO_4_ × 5H_2_O, and 36 μM FeEDTA. After 4–7 days, when seedlings had reached the 4-leaf stage, plants were sorted according to their root number and only those with 11–12 roots were used for the following experiments. The seeds were carefully removed and plants were transferred intact (Experiment 2) or after having cut off roots just below the crown (Experiment 3). Plants were transferred to 12 L boxes containing agar nutrient solution (0.1% agar in full strength Yoshida solution), as described earlier (Wang et al., 2008). Bicarbonate (1 mM KHCO_3_) was added to buffer the pH at 7.5–7.9, which is typical for paddy fields, like the one used in Experiment 1. Two Zn treatments were imposed, either –Zn (no Zn) or a +Zn control (1.5 μM ZnSO_4_ × 7H_2_O). The nutrient solution was renewed every week. Up to 23 plants per box were grown for 2 weeks. At 0 and 2 weeks after transfer (WAT), roots were counted and plants were harvested to determine root and shoot dry weight and Zn content. Deionized water was used for nutrient solutions during the pre-treatment phase. Solutions for the treatments were prepared using ultrapure water (YamaSakae purified water, Japan) to avoid any Zn contamination.

### Tissue Digestion and Zn Measurements

Dry shoot and root tissue were cut into small pieces with scissors. Seeds were ground in a mortar using a pestle. Approximately 100 mg tissue was used for shoot and seeds and 30 mg for roots; when less was available, the total sample amount was used. Samples were microwave-digested in a mix of 2 mL 30% hydrogen peroxide and 0.5 mL concentrated metal grade nitric acid, according to Miller (1998). All containers had been acid washed in 1 M HCl prior to digestion and ultrapure water (YamaSakae purified water, Japan) was used for dilutions. The concentration of Zn in the digest was determined by ICP-AES (ICPE-900, Shimadzu, Japan).

### Histological Analysis

Crowns were harvested 2 WAT to –Zn and +Zn conditions of Experiment 2 and fixed in 4% formamide in PBS buffer overnight, before dehydration through a graded ethanol series. Samples were embedded in Paraplast Plus (Leica Biosystems, Japan) and 10 μm sections were prepared using a rotary-microtome. Sections were stained using a 0.05% toluidine blue solution and observed with an Olympus BX50 light microscope (Olympus, Tokyo, Japan), fitted with an Olympus DP21 digital camera.

### Statistical Analysis

All statistical analyses were done using the Statistix 9 software (Analytical Software, Tallahassee, FL, USA). Statistical significance between measurements was determined using Tukey’s honest significant difference (HSD) all pair-wise comparisons.

## Results

### Experiment 1

#### Root Growth and Biomass Accumulation under Zn Deficiency in the field

**FIGURE 1:**
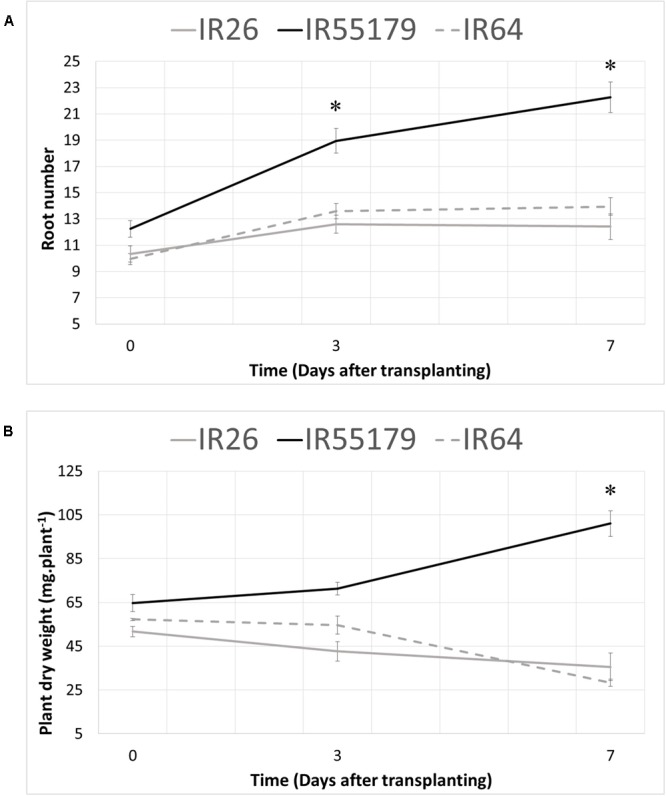
**Root number and total plant dry weight 0, 3, and 7 days after transplanting to a Zinc (Zn) deficient field.** Zn-efficient, IR55179, and Zn-inefficient, IR26 and IR64, plants were harvested at several time points and total root number **(A)** and total plant dry weight **(B)** were assessed. Statistical significant differences between genotypes (*p* < 0.05) are indicated by ^∗^. Error bars represent SEM (*n* = 5).

In order to investigate the response to Zn deficiency at the seedling stage, root development and biomass accumulation were investigated in low-Zn field conditions (available Zn content 0.1 μg g^-1^, Izquierdo et al., 2016). For this, one Zn-efficient, IR55179, and two Zn-inefficient, IR26 and IR64, genotypes were used. Root number and whole plant dry weight were determined at 0, 3, and 7 DAT (**Figure 1**). On the day of transplanting (0 DAT) Zn-efficient IR55179 already had a higher number of roots (**Figure 1A**) and plant dry weight (**Figure 1B**), compared to the Zn-inefficient genotypes IR26 and IR64. At 3 DAT, the difference of response to Zn deficiency between the Zn-efficient and inefficient genotypes was already apparent: IR55179 showed a 55% increase in root number, with a slight increase in total dry weight of 10%, whereas IR26 and IR64 had only increased their root number by 22 and 37%, respectively (**Figure 1**). Moreover, total biomass of IR26 and IR64 actually decreased due to the loss of some older leaves following transplanting, which was not yet compensated by newly emerging leaves. At 7 DAT, root number of IR55179 had almost doubled and dry weight had increased by 56% relative to 0 DAT. IR26 and IR64, on the other hand, seemed arrested in their development, with little or no further increase in root number and continuously decreasing dry weight.

### Experiment 2

#### Root Growth and Biomass Accumulation under Severe Zn Deficiency in Nutrient Solution

**FIGURE 2:**
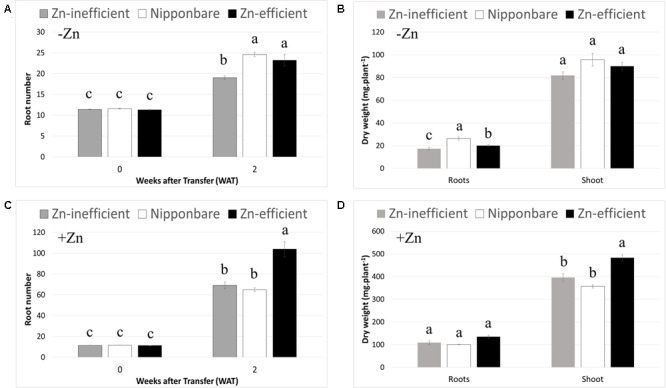
**Root number and root and shoot dry weight at 0 and 2 weeks after transfer (WAT) to nutrient solutions.** Nipponbare, Zn-efficient (IR55179 and RIL46) and Zn-inefficient (IR26, IR64, IR74) genotypes were transferred to nutrient solution with (+Zn) and without (-Zn) Zn. Root number **(A)** and root and shoot dry weights **(B)** in nutrient solution without Zn. Root number **(C)** and root and shoot dry weights **(D)** in nutrient solution with 1.5 μM Zn. Statistical significant differences (*p* < 0.05) are indicated by different letters within each time point or for each tissue. Error bars represent SEM (*n* = 3 per genotype).

To ascertain whether IR15579 and other Zn-efficient genotypes are Zn-efficient because they grow faster and start out bigger, when exposed to Zn deficiency, we selected seedlings with the same number of roots and leaves (11–12 roots and four leaves) at the beginning of the treatment (0 WAT). This selection resulted in plants with similar shoot and root dry weights before treatment and, therefore, a more relevant comparison of plant development between Zn-efficient and inefficient genotypes, in response to Zn deficiency (Supplementary Table S1). Moreover, to our knowledge, Nipponbare has never been reported to be either Zn-efficient or inefficient. Considering the available resources around Nipponbare and its wide use as reference genome, this knowledge could be of great interest to help uncover new candidate genes controlling Zn efficiency. Nipponbare was, therefore, included in this experiment. RIL46 and IR55179 were used as Zn-efficient and IR26, IR64, and IR74 were used as Zn-inefficient genotypes. In order to highlight differences between Zn-efficient and inefficient genotypes, in response to Zn-deficiency, data from individual genotypes was pooled into three groups: Zn-efficient, Zn-inefficient, and Nipponbare. However, data for individual genotypes can be found in the supplementary data (Supplementary Table S2). At 2 WAT to nutrient solution without Zn (–Zn), root number of Zn-efficient genotypes had increased by 112% (from 11.6 to 24.6), while Zn-inefficient genotypes only increased by 67% (from 11.4 to 19; **Figure 2A**). The increase in root number of Nipponbare was similar to the Zn-efficient genotypes (106%, from 11.3 to 23.2). Significant differences between genotypes were detected in root dry weight, but not in shoot dry weight (**Figure 2B**). Root biomass of Zn-inefficient genotypes had increased by 218% (from 5.43 to 17.3 mg) since 0 WAT, compared to 324% (from 4.72 to 20.1 mg) for Zn-efficient genotypes and 459% (from 4.71 to 26.3 mg) in Nipponbare (Supplementary Table S1 and **Figure 2B**).

It is noteworthy that Zn-efficient genotypes also developed more roots, compared to Zn-inefficient genotypes, when grown in nutrient solution containing Zn (+Zn; **Figure 2C**). Interestingly, in the presence of Zn, Nipponbare developed a similar number of roots as the Zn-inefficient genotypes, rather than follow the trend of the Zn-efficient genotypes. Zn-efficient genotypes also accumulated a higher shoot biomass in the presence of Zn, compared to Zn-inefficient genotypes and Nipponbare (**Figure 2D**). However, when comparing root number in -Zn with that in +Zn treatments, a decrease of 76% for Zn-efficient genotypes was observed, while the root number of Nipponbare and Zn-efficient genotypes only decreased by 64 and 72%, respectively (**Figures 2A,C**). Similarly, root and shoot biomass accumulation of Zn-efficient genotypes decreased by 85 and 81%, respectively, in -Zn treatments compared to +Zn, while that of Zn-inefficient genotypes decreased by 84% for root and 79% for shoot biomass (**Figures 2B,D**). Nipponbare performed the best in the absence of Zn, relative to when Zn was provided, with only a 74% decrease in root biomass and 73% for the shoot.

#### Zinc Retranslocation under Zinc Deficiency

**FIGURE 3:**
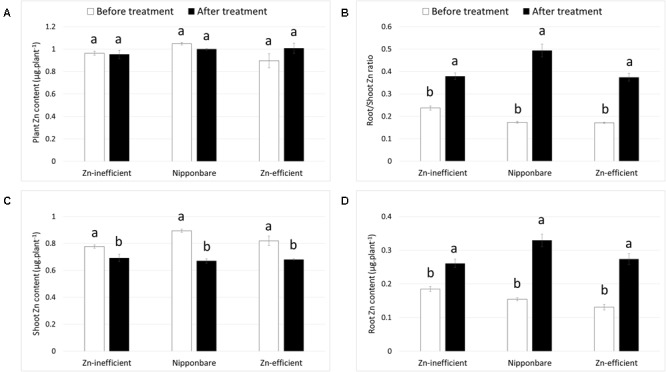
**Zinc content and retranslocation of Nipponbare, Zn-efficient and inefficient genotypes growing in nutrient solution without Zn.** Zn content in whole plants **(A)**, Zn root to shoot ratio **(B)** and Zn content of shoot **(C)** and roots **(D)** before (0 WAT) and after (2 WAT) growth without Zn. Statistical significant differences (*p* < 0.05) are indicated by different letter for each group. Error bars represent SEM (*n* = 3 per genotype).

Nipponbare and Zn-efficient genotypes were capable of developing more roots than Zn-inefficient genotypes in the complete absence of an external source of Zn. In order to accomplish this, Nipponbare and Zn-efficient genotypes must contain more Zn, be capable of growing more roots per unit of Zn available, or retranslocating more Zn from other tissues to the roots, or all of the above. To investigate this, Zn content of Nipponbare, Zn-efficient and inefficient genotypes was measured in the seed (before sowing) and in the shoot and the roots at different time points. Seeds from Zn-inefficient genotypes contained 22% less Zn than seeds from Zn-efficient genotypes (**Table 1**, Supplementary Table S3). Nipponbare seeds also contained less Zn, compared to those of Zn-efficient genotypes, but only by 9% (**Table 1**). However, at 0 WAT, seedlings of all genotypes contained the same total amount of Zn in their shoot and roots, though its distribution was slightly different (**Figures 3A,B** and Supplementary Table S4). Zn-inefficient genotypes had a higher proportion of Zn in the roots, resulting in a higher root to shoot ratio (0.24) at 0 WAT, compared to the Zn-efficient genotypes (0.17) and Nipponbare (0.17; **Figure 3B**). At 2 WAT to Zn-free nutrient solution, no difference in total Zn content was observed for either Nipponbare, Zn-inefficient or efficient genotypes, confirming that no Zn uptake took place (**Figure 3A**). However, during the 2-week-growth period, 17% of shoot-Zn was retranslocated to roots in Zn-efficient plants, compared to only 11% in Zn-inefficient plants (**Figure 3C**). Impressively, Nipponbare retranslocated 25% of its shoot-Zn to roots. This resulted in an increase in root-Zn of 113% for Nipponbare and 109% for Zn-efficient genotypes, while the root-Zn of Zn-inefficient genotypes only increased by 41% (**Figure 3D**). As a result, the Zn root to shoot ratio of Nipponbare and Zn-efficient genotypes increased by 185 and 118%, respectively, while that of Zn-inefficient genotypes only increased by 60% (**Figure 3B**).

**Table 1:** Zinc (Zn) content per dry seed of Nipponbare, Zn-efficient and inefficient genotypes.

*Item*	*Zn-inefficient*	*Nipponbare*	*Zn-efficient*
	(IR26, IR74, IR64)		(IR55179, RIL46)
Seed dry weight (mg seed^-1^)	21.7^a^	24.3^a^	24.1^a^
Seed Zn content (μg seed^-1^)	0.39^b^	0.46^ab^	0.50^a^

*Average values of Zn-inefficient and efficient genotypes are given. Statistical significant differences between values (*p* < 0.05) are indicated by different letters within each row (*n* = 3 per genotype).*

#### Crown Root Development under Zn Deficiency

**FIGURE 4:**
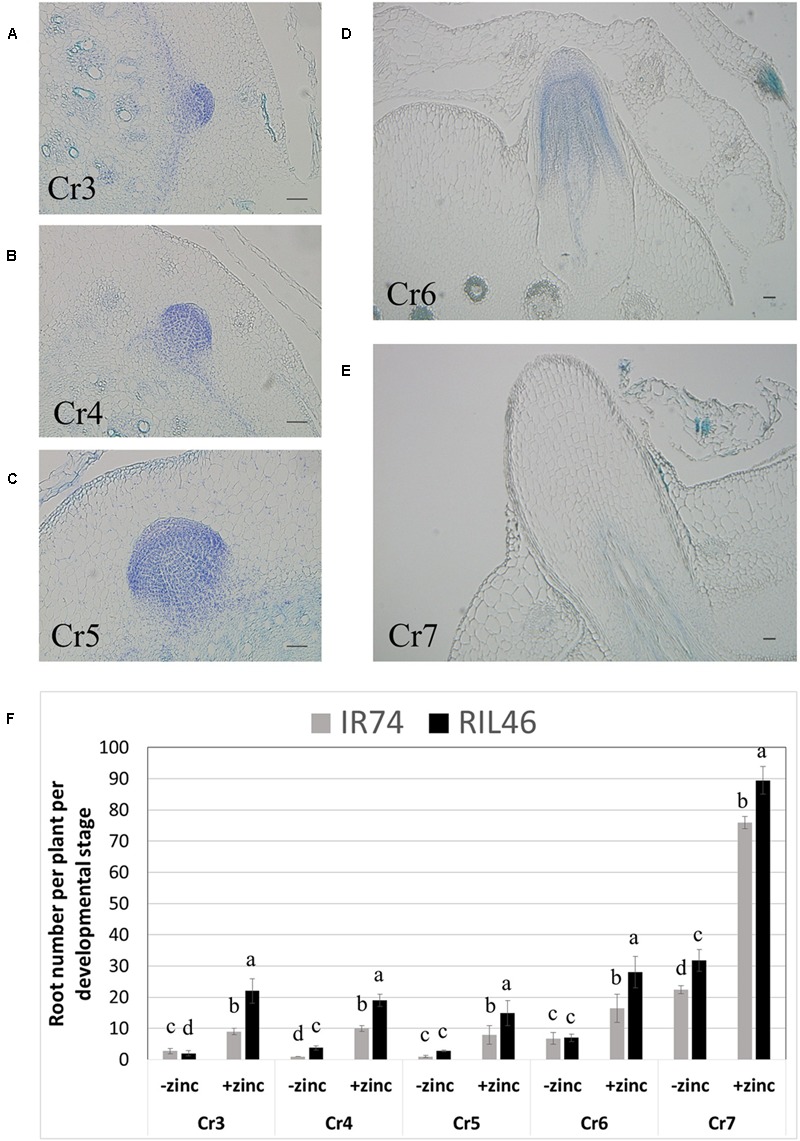
**Crown root development in IR74 (Zn-inefficient) and RIL46 (Zn-efficient), grown in nutrient solution with and without Zn.** Cross-sections of crowns showing crown root developmental stages from differentiation of the epidermis and endodermis, Cr3, to emergence, Cr7 **(A–E)**. Scale bar: 50 μm. Average number of crown roots at different developmental stages, from Cr3 to Cr7, in RIL46 and IR74, grown in nutrient solutions containing no (-zinc) or 1.5 μM Zn (+zinc) **(F)**. Statistical significant data (*p* < 0.05) between crown root developmental stages (Cr) are indicated by different letters. Error bars represent SEM (*n* = 5).

To investigate in more details the effects of Zn deficiency on root development, crowns were harvested 2 WAT, cross-sectioned and crown root developmental stages were observed microscopically. Seven stages of crown root development have been described, from the establishment of the initial cells that form the crown root primordia (Cr1), to the emergence of the crown root (Cr7; Itoh et al., 2005). Our technique allowed us to easily and reproducibly identify developing crown roots from stages Cr3 (differentiation of endodermis and epidermis) up to Cr7 (crown root emergence; **Figures 4A–E**). Although absolute crown root number per developmental stage varied between replicates of the same genotypes, the same trend was always observed: in the absence of Zn, crown roots from stages Cr3 to Cr6 were few and similar in both RIL46 and IR74 (**Figure 4F**). However, the total sum of developing roots (*t*) from stages Cr3 to Cr6 was 25% higher in the Zn-efficient RIL46 (*t* = 15.6), compared to the inefficient IR74 (*t* = 11.6). The number of emerged crown roots (stage Cr7) was higher than root number counts obtained from whole plants from the same experiment (**Figure 2A**), which was most likely due to roots that had emerged, but were still too short to be observed with the naked eye. Nevertheless, RIL46 had 30% more emerged roots (*t* = 31.8), compared to IR74 (*t* = 22.4). A similar trend was found when grown in the presence of Zn, only at a higher rate (**Figure 4F**).

### Experiment 3

#### Root Regrowth under Zn Deficiency

**FIGURE 5:**
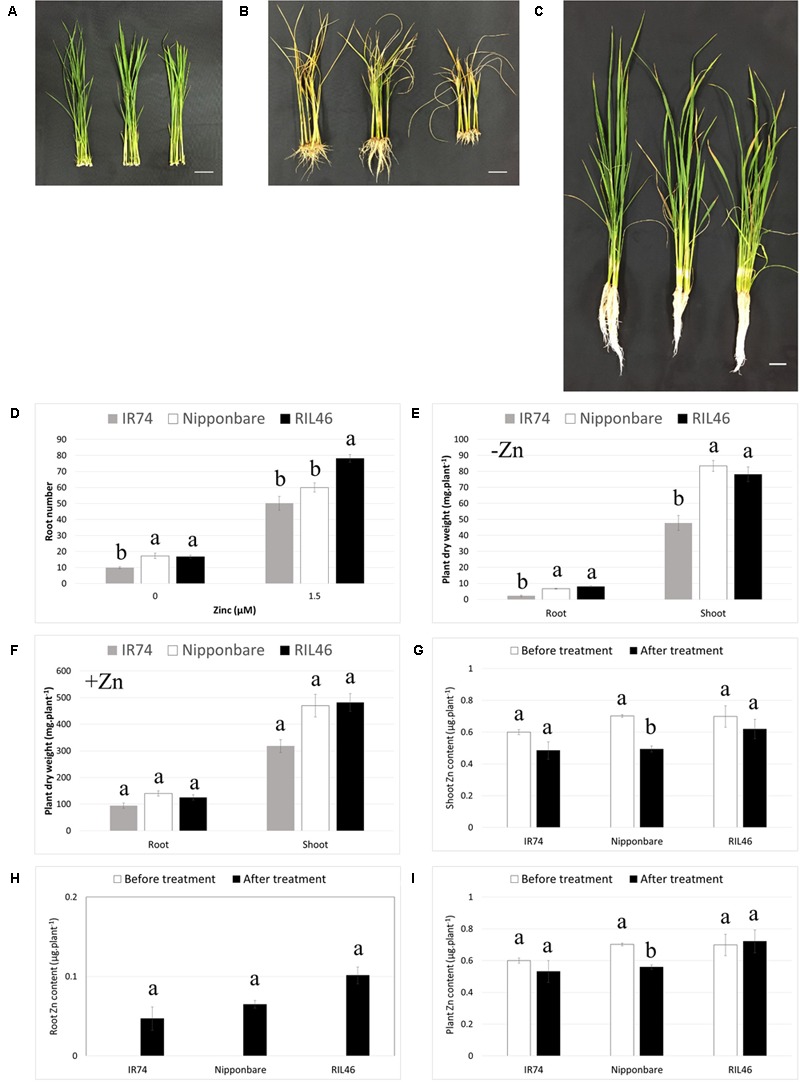
**Regeneration of roots 2 weeks after cutting and transferring to nutrient solution with and without Zn.** Nipponbare (left) and Zn-efficient RIL46 (middle), as well as Zn-inefficient IR74 (right), at 0 WAT **(A)**, 2 WAT to Zn-free nutrient solution **(B)** and 2 WAT to nutrient solution containing 1.5 μM Zn **(C)**. Scale bar: 3 cm. Root number (*n* = 6) **(D)** and root and shoot dry weights 2 WAT to nutrient solution containing 0 **(E)** and 1.5 μM **(F)** Zn (*n* = 2). Zn content in shoots **(G)** and roots **(H)** and whole plants **(I)** before (0 WAT) and after (2 WAT) growth without Zn. Statistical significant data (*p* < 0.05) are indicated by different letters for each genotype. Error bars represent SEM (*n* = 2).

During transplanting from the nursery bed to the field, rice roots are often damaged or broken and new roots need to grow after transplanting. Therefore, the ability of a genotype to regenerate its roots after cutting or damage could be an important aspect of Zn-efficiency. In order to investigate this, Zn-efficient RIL46, Nipponbare and Zn-inefficient IR74 plants were selected based on equal leaf and root number (four leaves and 11–12 roots). The attached seed was carefully removed and roots were cut right under the crown, before transferring plants to nutrient solution with or without Zn (**Figure 5A**). At that point plant biomass, made up by the crown and shoot, was similar between all genotypes (Supplementary Table S5). At 2 WAT to Zn-free nutrient solution, RIL46 and Nipponbare had regrown nearly twice the amount of roots, compared to IR74 (**Figures 5B,D**). IR74 also accumulated around 70 and 40% less root and shoot biomass, respectively, compared to Nipponbare and RIL46 (**Figure 5E**). However, IR74 also grew slightly less roots and accumulated less plant biomass, compared to RIL46, in the presence of Zn (**Figures 5C,D,F**). Similar to observations in Experiment 2, Nipponbare followed the same trend as IR74 in the presence of Zn, rather than that of RIL46, in regard to its root number (**Figure 5D**). However, its accumulated shoot biomass was closer to that of RIL46 than that of IR74 (**Figure 5F**). When comparing + and -Zn treatments, RIL46 presented a 79% decrease in root number, while Nipponbare and IR74 presented a decrease of 71 and 80%, respectively (**Figure 5D**). Root biomass in the –Zn treatment decreased by 94, 95, and 98% in RIL46, Nipponbare and IR74, respectively, compared to the +Zn treatment, while shoot biomass decreased by 84, 82, and 85%, respectively (**Figures 5E,F**).

#### Zinc Retranslocation for Root Regeneration

As observed in Experiment 2, IR74 contained the same total Zn content at 0 WAT, compared to Zn-efficient genotypes, but a higher Zn root to shoot ratio. This resulted in IR74 shoots containing 15% less Zn, compared to RIL46 and Nipponbare at 0 WAT (**Figure 5G**). All genotypes retranslocated Zn from the shoot to the root, in order to regrow roots after cutting. However, in IR74 plants, only 8% of the shoot-Zn at 0 WAT was retranslocated to roots, even though the decrease in shoot Zn was of 20% (**Figures 5G–I**). The same was observed in Nipponbare plants, 9% of shoot-Zn at 0 WAT was retranslocated to roots, while shoot-Zn content decreased by 30%. This loss of Zn was most likely due to Zn diffusing into the nutrient solution following wounding. Interestingly, at 2 WAT, RIL46 roots contained 14% of the shoot-Zn at 0 WAT, while the shoot-Zn had only decreased by 11%, suggesting that RIL46 might have taken up some of the Zn diffused into the nutrient solution by the other genotypes.

## Discussion

### Zn Deficiency Affects Crown Root Initiation Rather than Emergence

Previous reports have demonstrated decreased crown root development in rice under Zn deficiency (Widodo et al., 2010; Rose et al., 2013; Mori et al., 2016). However, how and at what stage Zn deficiency causes this decrease remained unknown. Microscopic observations in the present study showed fewer crown roots had been initiated under Zn deficiency at the first visible stage Cr3. The reduced number of crown root initiates carried through all subsequent stages (Cr3-Cr7). No accumulation of crown roots at any particular developmental stage was detected, nor did micrographs indicate any deformation or otherwise atypical development. Thus, no evidence suggests that crown root emergence was affected or even arrested by Zn deficiency. Instead, Zn deficiency appears to have affected the rate of crown root initiation. Further studies, specifically looking into the very first steps of crown root formation (stages Cr1 and Cr2) should be undertaken to confirm this.

In rice, crown root initiation is regulated by several genes, including the *CRL* genes, many of whose expression is regulated by auxin (Inukai et al., 2001a,b, 2005; Kitomi et al., 2008a,b, 2011). Previous reports state that Zn is involved in auxin metabolism (Marschner, 1995), but the mechanism remains unclear. Skoog (1940) demonstrated a severe decrease in auxin in tomato and sunflower, in response to Zn deficiency. Interestingly, this decrease preceded any visible symptoms of Zn deficiency and auxin increased a few days following Zn application (Skoog, 1940). Similarly, Zn deficient treatments of Bean (*Phaseolus vulgaris*) resulted in a reduction of indole-3-acetic acid (Cakmak et al., 1989). Finally, a gene expression study of a Zn-efficient and an inefficient genotype, exposed to Zn deficiency in the field, identified several auxin-related genes to be differentially expressed between the two (Widodo et al., 2010). However, no significant differences in expression levels of any of the *CRL* genes, included on the Affymetrix microarray, were found. This suggests that Zn deficiency affects crown root development through a parallel signaling pathway to that of the *CRL* genes, but one that might also be regulated by auxin signaling.

### Faster Crown Root Development: A Constitutive or Zn Deficiency-Specific Trait?

IR55179 and RIL46 have been widely studied for their tolerance to Zn deficiency, compared to IR26, IR64, and IR74, both in the field and in nutrient solution (Bowen, 1986; Quijano-Guerta et al., 2002; Xiaopeng Gao, 2005; Wissuwa et al., 2006; Widodo et al., 2010; Mori et al., 2016). However, to our knowledge, their growth phenotype in the absence of stress has not been reported in detail. Moreover, their Zn efficiency has typically been determined by root growth and biomass accumulation after exposure to Zn deficiency, without specifically taking pre-treatment performance, or performance under Zn sufficiency, into account. At the seedling to mid-tillering stage, growth is exponential and small genotypic differences in root number or plant biomass, at the time of transplanting, could be expanded later on. Moreover, bigger seedlings are likely to contain more Zn compared to smaller ones, resulting in plants with more Zn reserves available for retranslocation to overcome Zn deficiency. Here, we hypothesized that IR55179 and RIL46 might be Zn-efficient because of their head start before being exposed to Zn deficiency. While our data could not exclude the possibility that bigger seedlings did have a positive effect in the field, subsequent experiments with seedlings, selected to be of the same size and developmental stage for all genotypes, indicated that IR55179 and RIL46 developed more rapidly under Zn deficiency, compared to the inefficient genotypes. However, more rapid root and shoot development was not limited to Zn deficiency, but also manifested itself in +Zn control treatments, suggesting that a constitutive component also plays a role. In fact, our data demonstrated that Zn-efficient IR55179 and RIL46 actually presented the most severe decrease in root number and plant biomass accumulation, of any genotype used in this study, in the absence of Zn, compared to when Zn was provided. This raises the question of whether these genotypes should really be considered Zn-efficient, or not? However, one should keep in mind that the Zn deficiency treatments used in the nutrient solutions were completely devoid of Zn and did, therefore, not reflect realistic field conditions, where Zn is often present but poorly accessible to the plant (Dobermann and Fairhurst, 2000). The ability to take up poorly available Zn in the field was not investigated in this study and could be a key trait linked to the Zn-efficiency of these genotypes. Detailed studies of Zn-efficient IR55179 and RIL46, compared to Zn-inefficient genotypes, in both Zn deficient and sufficient fields should be undertaken to investigate this further.

### Genotypic Differences in Zn Translocation and Zn Utilization Efficiency

Several studies have investigated Zn translocation and remobilisation during rice growth and in response to Zn deficiency. However, they mostly focused on more mature plants and grain filling, rather than early vegetative growth (Wu et al., 2010; Impa et al., 2013). In transplanted rice, most roots are severely damaged during the uprooting of seedlings and subsequent transplanting, resulting in a ‘transplanting shock.’ First signs of Zn deficiency usually appear 1 week after transplanting and the following 2 to 3 weeks are most crucial in overcoming this stress. This would correspond to the period after which plants have overcome the stress of transplanting and resumed normal growth in the absence of Zn deficiency. Here, we show that when Zn was not limiting, Zn was taken up by the roots, as well as mobilized and translocated from the seed to the seedling. Interestingly, genotypes with high seed Zn content did not give seedlings with higher Zn content than genotypes with low seed Zn. Moreover, in our experiments, seedlings started taking up Zn from the nutrient solution while some was still remaining in their seed (Supplementary Table S6). This suggests that some seed Zn might be stored after germination, possibly to overcome a Zn limitation later on, or that it is not as readily available to the growing seedlings, as the Zn in nutrient solution. This is in accordance with other studies demonstrating that seed Zn content does not affect Zn efficiency of the future plant (Xiaopeng Gao, 2005; Impa et al., 2013).

Zinc is involved in enzyme activation, protein synthesis and photosynthesis, but also in the metabolism of lipids, carbohydrates, and amino acids (Marschner, 1995). Therefore, accumulating Zn in the shoot will allow its rapid and uninhibited growth. The products of these processes can then be translocated to the roots, to ensure root growth can meet up to the needs of the ever growing shoot. However, in the absence of an external source of Zn, as in our Zn-free nutrient solutions, the plants retranslocated Zn from the shoot to the roots, thereby switching their source-sink roles with regard to Zn. The fact that Zn-efficient IR55179 and RIL46 retranslocated a higher proportion of Zn from their shoot to roots, compared to Zn-inefficient genotypes, suggests they were better in prioritizing root growth under Zn deficiency, which is of particular importance following the loss of functional roots in the transplanting process. These findings suggest that the mechanisms by which these Zn-efficient genotypes overcome Zn deficiency could be a combination of better constitutive growth and better Zn utilization efficiency (ZUE).

### Nipponbare is Zn-Efficient in a Zn-Specific Manner

To our knowledge, Nipponbare had never been assessed for its Zn-efficiency. Here, we demonstrated that Nipponbare was capable of developing crown roots and accumulating root biomass under severe Zn deficiency, at the same rate as other well-known Zn-efficient genotypes. This was achieved, at least partly, through efficient retranslocation of shoot-Zn to roots, in order to maintain crown root development under severe Zn deficiency. Moreover, in the absence of an external source of Zn, Nipponbare was also capable of regrowing its roots, after cutting, to a similar extent as the Zn-efficient RIL46. Interestingly, while RIL46 retranslocated a higher proportion of its shoot-Zn for this purpose, Nipponbare achieved this by retranslocating a similar proportion of its shoot-Zn, as what was observed for the Zn-inefficient IR74. This suggests that Nipponbare is more efficient in its use of Zn to generate new roots, compared to IR74 and, possibly even, RIL46. Moreover, since IR74 was not capable of regrowing its roots to the same extent as Nipponbare, even though both retranslocated the same amount of Zn to their roots (**Figure 5**), other factors need to be considered and may have played an inhibitory role in root growth, that reduced the internal ZUE in susceptible genotypes, like IR74. Further studies will need to be conducted to investigate, in detail, the mechanisms of Zn-efficiency of Nipponbare, compared to other Zn-efficient genotypes, as well as to clarify the role of auxin and/or potential inhibitory factors in these processes.

Nipponbare was the only Zn-efficient genotype that did not display a constitutive, but rather a Zn deficiency-specific advantage under our conditions. Nipponbare maintained crown root development and plant biomass accumulation to a higher extent than any other genotype used in this study. Since the sequencing of its genome (International Rice Genome Sequencing Project, 2005), Nipponbare has become the reference genome against which all other rice cultivars are compared. As a result, it is ideal for the identification of candidate genes conferring Zn-efficiency through the maintenance of crown root development. However, whether Nipponbare’s Zn efficiency will hold true in the field remains unknown.

### Maintenance of Crown Root Development is Key in Overcoming Zn Deficiency

In the field, a difference in crown root number between Zn-efficient and inefficient genotypes was already apparent 3 DAT to Zn deficient soil, underlining the importance of an early response to overcome Zn deficiency. At the same time, plant biomass was not much affected, demonstrating that the maintenance of crown root development is the first visible manifestation of Zn efficiency and one of the principle underlying causes, rather than being a consequence of better Zn acquisition, due to hypothesized rhizosphere mechanisms, such as exudation of compounds enhancing Zn bioavailability (Zhang et al., 1991; Suzuki et al., 2008; Ptashnyk et al., 2011). This is further supported in nutrient solution in the absence of Zn, where crown root number and root biomass, but not shoot biomass, was higher in Zn-efficient genotypes, compared to the Zn-inefficient ones. This trait could constitute a fast and reliable method to screen for Zn efficiency for future breeding. Interestingly, no correlation between maximum root length and Zn-efficiency was found (data not shown), suggesting that root number, rather than root length, drives Zn-efficiency.

## Conclusion

To prevent yield losses associated with Zn deficiency in rice, crop establishment following transplanting is important and the rapid development of a root system to secure new Zn uptake is a key factor in this regard. Zn-efficient genotypes are capable of doing so through a combination of factors that include (i) prioritizing of root growth by translocating more Zn and, possibly, other compounds from the shoot to the root; (ii) better utilization of Zn in root development, resulting in more roots developed for a same amount of available Zn; and (iii) constitutive high seedling vigor that may lead to the accumulation of more resources, including Zn, in the pre-transplanting period.

## Author Contributions

Undertook experiments and analyzed data: AN. Scientific discussions and writing of the manuscript: AN and MW.

## Conflict of Interest Statement

The authors declare that the research was conducted in the absence of any commercial or financial relationships that could be construed as a potential conflict of interest.

## References

[B1] Bowen J. E. (1986). Kinetics of zinc uptake by two rice cultivars. *Plant Soil* 94 99–107. 10.1007/BF02380592

[B2] Breemen N. V. Castro R. U. (1980). Zinc deficiency in wetland rice along a toposequence of hydromorphic soils in the Philippines. *Plant Soil* 57 215–221. 10.1007/BF02211681

[B3] Cakmak I. Marschner H. Bangerth F. (1989). Effect of zinc nutritional status on growth, protein metabolism and levels of indole-3-acetic acid and other phytohormones in bean (*Phaseolus vulgaris* L.). *J. Exp. Bot.* 40 405–412. 10.1093/jxb/40.3.405

[B4] Coudert Y. Périn C. Courtois B. Khong N. G. Gantet P. (2010). Genetic control of root development in rice, the model cereal. *Trends Plant Sci.* 15 219–226. 10.1016/j.tplants.2010.01.008 20153971

[B5] Dobermann A. Fairhurst T. (2000). *Rice: Nutrient Disorders & Nutrient Management.* Makati City: Potash & Phosphate Institute, East & Southeast Asia Programs.

[B6] Food and Agriculture Organization (2015). *State of food Insecurity in the World 2015.* Rome: Food and Agriculture Organization.

[B7] Gamuyao R. Chin J. H. Pariasca-Tanaka J. Pesaresi P. Catausan S. Dalid C. (2012). The protein kinase Pstol1 from traditional rice confers tolerance of phosphorus deficiency. *Nature* 488 535–539. 10.1038/nature11346 22914168

[B8] Hoshikawa K. (1989). *The Growing Rice Plant: An Anatomical Monograph.* Tokyo: Nobunkyo.

[B9] Impa S. M. Gramlich A. Tandy S. Schulin R. Frossard E. Johnson-Beebout S. E. (2013). Internal Zn allocation influences Zn deficiency tolerance and grain Zn loading in rice (*Oryza sativa* L.). *Front. Plant Sci.* 4:534 10.3389/fpls.2013.00534 PMC3871718 24400015

[B10] Inukai Y. Miwa M. Nagato Y. Kitano H. Yamauchi A. (2001a). Characterization of rice mutants deficient in the formation of crown roots. *Breed. Sci.* 51 123–129. 10.1270/jsbbs.51.123

[B11] Inukai Y. Miwa M. Nagato Y. Kitano H. Yamauchi A. (2001b). RRL1, RRL2 and CRL2 loci regulating root elogation in rice. *Breed. Sci.* 51 231–239. 10.1270/jsbbs.51.231

[B12] Inukai Y. Sakamoto T. Ueguchi-Tanaka M. Shibata Y. Gomi K. Umemura I. (2005). Crown rootless1, which is essential for crown root formation in rice, is a target of an AUXIN RESPONSE FACTOR in Auxin Signaling. *Plant Cell* 17 1387–1396. 10.1105/tpc.105.030981 15829602 PMC1091762

[B13] Ismail A. M. Heuer S. Thomson M. J. Wissuwa M. (2007). Genetic and genomic approaches to develop rice germplasm for problem soils. *Plant Mol. Biol.* 65 547–570. 10.1007/s11103-007-9215-2 17703278

[B14] Itoh J.-I. Nonomura K.-I. Ikeda K. Yamaki S. Inukai Y. Yamagishi H. (2005). Rice Plant Development: from zygote to spikelet. *Plant Cell Physiol.* 46 23–47. 10.1093/pcp/pci501 15659435

[B15] Izquierdo M. Impa S. M. Johnson-Beebout S. E. Weiss D. J. Kirk G. J. D. (2016). Measurement of isotopically-exchangeable Zn in Zn-deficient paddy soil. *Eur. J. Soil Sci.* 67 51–59. 10.1111/ejss.12303

[B16] Johnson-Beebout S. E. Lauren J. G. Duxbury J. M. (2009). Immobilization of zinc fertilizer in flooded soils monitored by adapted DTPA soil test. *Commun. Soil Sci. Plant Anal.* 40 1842–1861. 10.1080/00103620902896738

[B17] Kitomi Y. Ito H. Hobo T. Aya K. Kitano H. Inukai Y. (2011). The auxin responsive AP2/ERF transcription factor CROWN ROOTLESS5 is involved in crown root initiation in rice through the induction of OsRR1, a type-A response regulator of cytokinin signaling. *Plant J.* 67 472–484. 10.1111/j.1365-313X.2011.04610.x 21481033

[B18] Kitomi Y. Kitano H. Inukai Y. (2008a). Mapping of the CROWN ROOTLESS3 gene. CRL3, in rice. *Rice Genet. Newsl.* 24 31–33.

[B19] Kitomi Y. Ogawa A. Kitano H. Inukai Y. (2008b). CRL4 regulates crown root formation through auxin transport in rice. *Plant Root* 2 19–28. 10.3117/plantroot.2.19

[B20] Liu H. Wang S. Yu X. Yu J. He X. Zhang S. (2005). ARL1, a LOB-domain protein required for adventitious root formation in rice. *Plant J.* 43 47–56. 10.1111/j.1365-313X.2005.02434.x 15960615

[B21] Lynch J. P. (2007). Roots of the second green revolution. *Aust. J. Bot.* 55 493–512. 10.1071/BT06118

[B22] Mai C. D. Phung N. T. To H. T. Gonin M. Hoang G. T. Nguyen K. L. (2014). Genes controlling root development in. *Rice* 7:30 10.1186/s12284-014-0030-5 PMC4884052 26224559

[B23] Marschner H. (1995). *Mineral Nutrition of Higher Plants.* Boston: Academic Press.

[B24] Miller R. O. (1998). “Microwave digestion of plant tissue in an open vessel,” in *Handbook Reference Methods Plant Analysis* ed. Kalra P (Boca Raton, FL: CRC Press) 69–73.

[B25] Mori A. Kirk G. J. D. Lee J.-S. Morete M. J. Nanda A. K. Johnson-Beebout S. E. (2016). Rice genotype differences in tolerance of zinc-deficient soils: evidence for the importance of root-induced changes in the rhizosphere. *Front. Plant Sci.* 6:1160 10.3389/fpls.2015.01160 PMC4707259 26793198

[B26] Nene Y. (1966). Symptoms, cause and control of Khaira disease of paddy. *Bull Indian Phytopathol.* 3 97–101.

[B27] International Rice Genome Sequencing Project (2005). The map-based sequence of the rice genome. *Nature* 436 793–800. 10.1038/nature03895 16100779

[B28] Ptashnyk M. Roose T. Jones D. L. Kirk G. J. D. (2011). Enhanced zinc uptake by rice through phytosiderophore secretion: a modelling study. *Plant Cell Environ.* 34 2038–2046. 10.1111/j.1365-3040.2011.02401.x 21777252

[B29] Quijano-Guerta C. Kirk G. J. D. Portugal A. M. Bartolome V. I. McLaren G. C. (2002). Tolerance of rice germplasm to zinc deficiency. *Field Crops Res.* 76 123–130. 10.1016/S0378-4290(02)00034-5

[B30] Rebouillat J. Dievart A. Verdeil J. L. Escoute J. Giese G. Breitler J. C. (2009). Molecular Genetics of rice root development. *Rice* 2 15–34. 10.1007/s12284-008-9016-5

[B31] Rose T. J. Impa S. M. Rose M. T. Pariasca-Tanaka J. Mori A. Heuer S. (2013). Enhancing phosphorus and zinc acquisition efficiency in rice: a critical review of root traits and their potential utility in rice breeding. *Ann. Bot.* 112 331–345. 10.1093/aob/mcs217 23071218 PMC3698374

[B32] Rose T. J. Wissuwa M. (2012). “Chapter five - rethinking internal phosphorus utilization efficiency: a new approach is needed to improve PUE in grain crops,” in *Advances in Agronomy* ed. Sparks D. L. (Cambridge, MA: Academic Press) 185–217.

[B33] Sillanpää M. (1990). “Micronutrient Assessment at the Country Level: An International Study.” *FAO Soils Bulletin No. 63*. Rome: Food and Agriculture Organization 10.1186/s12889-016-2765-y

[B34] Skoog F. (1940). Relationships between zinc and auxin in the growth of higher plants. *Am. J. Bot.* 27 939–951. 10.2307/2436564

[B35] Suzuki M. Tsukamoto T. Inoue H. Watanabe S. Matsuhashi S. Takahashi M. (2008). Deoxymugineic acid increases Zn translocation in Zn-deficient rice plants. *Plant Mol. Biol.* 66 609–617. 10.1007/s11103-008-9292-x 18224446 PMC2268730

[B36] Uga Y. Sugimoto K. Ogawa S. Rane J. Ishitani M. Hara N. (2013). Control of root system architecture by DEEPER ROOTING 1 increases rice yield under drought conditions. *Nat. Genet.* 45 1097–1102. 10.1038/ng.2725 23913002

[B37] Wang Y. Frei M. Wissuwa M. (2008). An agar nutrient solution technique as a screening tool for tolerance to zinc deficiency and iron toxicity in rice. *Soil Sci. Plant Nutr.* 54 744–750. 10.1111/j.1747-0765.2008.00302.x

[B38] Widodo B. Broadley M. R. Rose T. Frei M. Pariasca-Tanaka J. Yoshihashi T. (2010). Response to zinc deficiency of two rice lines with contrasting tolerance is determined by root growth maintenance and organic acid exudation rates, and not by zinc-transporter activity. *New Phytol.* 186 400–414. 10.1111/j.1469-8137.2009.03177.x 20100202

[B39] Wissuwa M. Ismail A. M. Graham R. D. (2007). Rice grain zinc concentrations as affected by genotype, native soil-zinc availability, and zinc fertilization. *Plant Soil* 306 37–48. 10.1007/s11104-007-9368-4

[B40] Wissuwa M. Ismail A. M. Yanagihara S. (2006). Effects of zinc deficiency on rice growth and genetic factors contributing to tolerance. *Plant Physiol.* 142 731–741. 10.1104/pp.106.085225 16905666 PMC1586055

[B41] Wu C. Lu L. Yang X. Feng Y. Wei Y. Hao H. (2010). Uptake, translocation, and remobilization of zinc absorbed at different growth stages by rice genotypes of different zn densities. *J. Agric. Food Chem.* 58 6767–6773. 10.1021/jf100017e 20481473

[B42] Wu W. Cheng S. (2014). Root genetic research, an opportunity and challenge to rice improvement. *Field Crops Res.* 165 111–124. 10.1016/j.fcr.2014.04.013

[B43] Xiaopeng Gao C. Z. (2005). Tolerance to zinc deficiency in rice correlates with zinc uptake and translocation. *Plant Soil* 278 253–261. 10.1007/s11104-005-8674-y

[B44] Yoshida S. Ahn J. S. Forno D. A. (1973). Occurrence, diagnosis, and correction of zinc deficiency of lowland rice. *Soil Sci. Plant Nutr.* 19 83–93. 10.1080/00380768.1973.10432522

[B45] Yoshida S. Forno D. A. Cock J. H. Gomez K. A. (1972). *Laboratory Manual for Physiological Studies of Rice.* Los Baños: International Rice Research Institute.

[B46] Yoshida S. Tanaka A. (1969). Zinc deficiency of the rice plant in calcareous soils. *Soil Sci. Plant Nutr.* 15 75–80. 10.1080/00380768.1969.10432783

[B47] Zhang F. Romheld V. Marschner H. (1991). Release of zinc mobilizing root exudates in different plant species as affected by zinc nutritional status. *J. Plant Nutr.* 14 675–686. 10.1080/01904169109364234

