# Supplementary material for: Rapid Crown Root Development Confers Tolerance to Zinc Deficiency in Rice
Source: Front Plant Sci. 2016 Mar 31;7:428. doi: 10.3389/fpls.2016.00428 (PMC4815024; doi:10.3389/fpls.2016.00428)
Supplement: Supplementary file 1 [file Table_1.DOCX]

Supplementary Material

**Rapid crown root development confers tolerance to zinc deficiency in rice**

**Amrit K. Nanda, Matthias Wissuwa***

***Corresponding Author:** Matthias Wissuwa: [wissuwa@affrc.go.jp](mailto:nanda@affrc.go.jp)

**Table S1.** Experiment 2: Root number and plant dry weight of individual genotypes, before + and –Zn treatments (0 WAT). Statistical significant differences between genotypes (p < 0.05) are indicated by different letters within each row. The grouped averages of each value for Zn-efficient and inefficient genotypes are given in the following row (n = 3).

| Item | Zn-inefficient | | | Nipponbare | Zn-efficient | |
| --- | --- | --- | --- | --- | --- | --- |
|  | IR26 | IR74 | IR64 | Nipponbare | IR55179 | RIL46 |
| Total root number | 11.6 ^a^ | 11.2^a^ | 11.4^a^ | 11.3^a^ | 11.6^a^ | 11.6^a^ |
|  | 11.4^a^ | | | 11.5^a^ | | |
| Shoot DW (mg.plant^-1^) | 20.1^ab^ | 20.2^ab^ | 22.0^a^ | 20.1^ab^ | 22.2^a^ | 17.8^b^ |
|  | 20.8^a^ | | | 20.0^a^ | | |
| Root DW (mg.plant^-1^) | 5.33 ^ab^ | 4.99 ^ab^ | 5.96 ^a^ | 4.71^bc^ | 5.58^ab^ | 3.88^c^ |
|  | 5.43^a^ | | | 4.72^a^ | | |
