# Supplementary material for: Rapid Crown Root Development Confers Tolerance to Zinc Deficiency in Rice
Source: Front Plant Sci. 2016 Mar 31;7:428. doi: 10.3389/fpls.2016.00428 (PMC4815024; doi:10.3389/fpls.2016.00428)
Supplement: Supplementary file 2 [file Table_2.DOCX]

Supplementary Material

**Rapid crown root development confers tolerance to zinc deficiency in rice**

**Amrit K. Nanda, Matthias Wissuwa***

***Corresponding Author:** Matthias Wissuwa: [wissuwa@affrc.go.jp](mailto:nanda@affrc.go.jp)

**Table S2.** Experiment 2: Root number and plant dry weight of individual genotypes, after treatments with (+Zn) and without (-Zn) Zn (2 WAT). Statistical significant differences between genotypes (p < 0.05) are indicated by different letters within each row (n = 3).

| Item | Zn-inefficient | | | Nipponbare | Zn-efficient | |
| --- | --- | --- | --- | --- | --- | --- |
|  | IR26 | IR74 | IR64 | Nipponbare | IR55179 | RIL46 |
| *-Zn* |  |  |  |  |  |  |
| Total root number | 21^ab^ | 18.2^b^ | 17.9^b^ | 23.2^a^ | 24.6^a^ | 24.6^a^ |
| Shoot DW (mg.plant^-1^) | 86.6^a^ | 83.1^a^ | 75.9^a^ | 95.7^a^ | 86.4^a^ | 93.3^a^ |
| Root DW (mg.plant^-1^) | 16.7^b^ | 18.1^b^ | 17^b^ | 26.3^a^ | 20.6^b^ | 19.6^b^ |
| +Zn |  |  |  |  |  |  |
| Total root number | 56.5^d^ | 77.3^bc^ | 73.5^bc^ | 65^cd^ | 122^a^ | 85.8^b^ |
| Shoot DW (mg.plant^-1^) | 330.9^c^ | 434.2^a^ | 422.1^ab^ | 356.4^bc^ | 483.9^a^ | 481.4^a^ |
| Root DW (mg.plant^-1^) | 84.6^d^ | 137.4^ab^ | 104.1^bcd^ | 101.1^cd^ | 143^a^ | 126.8^abc^ |
