# Supplementary material for: Rapid Crown Root Development Confers Tolerance to Zinc Deficiency in Rice
Source: Front Plant Sci. 2016 Mar 31;7:428. doi: 10.3389/fpls.2016.00428 (PMC4815024; doi:10.3389/fpls.2016.00428)
Supplement: Supplementary file 3 [file Table_3.DOCX]

Supplementary Material

**Rapid crown root development confers tolerance to zinc deficiency in rice**

**Amrit K. Nanda, Matthias Wissuwa***

***Corresponding Author:** Matthias Wissuwa: [wissuwa@affrc.go.jp](mailto:nanda@affrc.go.jp)

**Table S3.** Experiments 2 and 3: Weight and Zn content of dry seeds from individual genotypes, before sowing.

| Item | Zn-inefficient | | | Nipponbare | Zn-efficient | |
| --- | --- | --- | --- | --- | --- | --- |
|  | IR26 | IR74 | IR64 | Nipponbare | IR55179 | RIL46 |
| Seed weight (mg.seed^-1^) | 19.7^c^ | 20.1^c^ | 25.2^b^ | 24.3^b^ | 28.6^a^ | 19.5^c^ |
| Zn content (μg.seed^-1^) | 0.34^c^ | 0.42^abc^ | 0.41^bc^ | 0.46^ab^ | 0.49^ab^ | 0.52^a^ |
