# Supplementary material for: Rapid Crown Root Development Confers Tolerance to Zinc Deficiency in Rice
Source: Front Plant Sci. 2016 Mar 31;7:428. doi: 10.3389/fpls.2016.00428 (PMC4815024; doi:10.3389/fpls.2016.00428)
Supplement: Supplementary file 4 [file Table_4.DOCX]

Supplementary Material

**Rapid crown root development confers tolerance to zinc deficiency in rice**

**Amrit K. Nanda, Matthias Wissuwa***

***Corresponding Author:** Matthias Wissuwa: [wissuwa@affrc.go.jp](mailto:nanda@affrc.go.jp)

**Table S4.** Experiment 2: Zn content and root to shoot ratio of individual genotypes before (0 WAT) and after –Zn treatment (2 WAT). Statistical significant differences (p < 0.05) are indicated by a different letter within each row (n = 3).

| Item | Zn-inefficient | | | Nipponbare | Zn-efficient | |
| --- | --- | --- | --- | --- | --- | --- |
|  | IR26 | IR74 | IR64 | Nipponbare | IR55179 | RIL46 |
| *0 WAT* |  |  |  |  |  |  |
| Plant Zn (μg.plant^-1^) | 0.98^ab^ | 0.93^ab^ | 0.98^ab^ | 1.05^a^ | 1.02^a^ | 0.81^b^ |
| Root Zn (μg.plant^-1^) | 0.18^ab^ | 0.17^ab^ | 0.21^a^ | 0.15^bc^ | 0.15^bc^ | 0.12^c^ |
| Shoot Zn (μg.plant^-1^) | 0.80^ab^ | 0.76^ab^ | 0.77^ab^ | 0.89^a^ | 0.88^a^ | 0.69^b^ |
| Zn Root/ Shoot ratio | 0.22^b^ | 0.22^b^ | 0.27^a^ | 0.17^c^ | 0.17^c^ | 0.17^c^ |
| 2 WAT |  |  |  |  |  |  |
| Plant Zn (μg.plant^-1^) | 1.03^a^ | 0.90^a^ | 0.92^a^ | 1.00^a^ | 1.07^a^ | 0.95^a^ |
| Root Zn (μg.plant^-1^) | 0.27^a^ | 0.25^a^ | 0.27^a^ | 0.33^a^ | 0.28^a^ | 0.26^a^ |
| Shoot Zn (μg.plant^-1^) | 0.77^a^ | 0.65^a^ | 0.65^a^ | 0.67^a^ | 0.78^a^ | 0.68^a^ |
| Zn Root/ Shoot ratio | 0.35^b^ | 0.38^ab^ | 0.41^ab^ | 0.49^a^ | 0.36^b^ | 0.39^ab^ |
