# Supplementary material for: Rapid Crown Root Development Confers Tolerance to Zinc Deficiency in Rice
Source: Front Plant Sci. 2016 Mar 31;7:428. doi: 10.3389/fpls.2016.00428 (PMC4815024; doi:10.3389/fpls.2016.00428)
Supplement: Supplementary file 5 [file Table_5.DOCX]

Supplementary Material

**Rapid crown root development confers tolerance to zinc deficiency in rice**

**Amrit K. Nanda, Matthias Wissuwa***

***Corresponding Author:** Matthias Wissuwa: [wissuwa@affrc.go.jp](mailto:nanda@affrc.go.jp)

**Table S5.** Experiment 3: Shoot dry weight, after cutting roots, before + or - Zn treatments (0 WAT). Statistical significant differences between values (p < 0.05) are indicated by different letters (n = 2).

| *Item* | IR74 | RIL46 | Nipponbare |
| --- | --- | --- | --- |
| Shoot DW (mg.plant^-1^) | 30.0^a^ | 35.5^a^ | 32.5^a^ |
