# Supplementary material for: Rapid Crown Root Development Confers Tolerance to Zinc Deficiency in Rice
Source: Front Plant Sci. 2016 Mar 31;7:428. doi: 10.3389/fpls.2016.00428 (PMC4815024; doi:10.3389/fpls.2016.00428)
Supplement: Supplementary file 6 [file Table_6.DOCX]

Supplementary Material

**Rapid crown root development confers tolerance to zinc deficiency in rice**

**Amrit K. Nanda, Matthias Wissuwa***

***Corresponding Author:** Matthias Wissuwa: [wissuwa@affrc.go.jp](mailto:nanda@affrc.go.jp)

**Table S6.** Experiment 2: Dry weight and zinc concentration and content of germinated seeds from 2 week-old plants, before + or – Zn treatments (0 WAT). Statistical significant differences between values (p < 0.05) are indicated by different letters within each row (n = 3).

| Item | Zn-inefficient | | Zn-efficient | |
| --- | --- | --- | --- | --- |
|  | IR26 | IR74 | IR55179 | RIL46 |
| Seed dry weight (mg.seed^-1^) | 5.60^a^ | 6.50^b^ | 8.15^c^ | 6.77^b^ |
| Seed zinc concentration (ppm) | 15.8^a^ | 13.1^a^ | 14.6^a^ | 31.1^b^ |
| Seed zinc content (μg.seed^-1^) | 0.09^a^ | 0.08^a^ | 0.12^a^ | 0.21^b^ |
